# Stomatal clustering in *Begonia* associates with the kinetics of leaf gaseous exchange and influences water use efficiency

**DOI:** 10.1093/jxb/erx072

**Published:** 2017-03-28

**Authors:** Maria Papanatsiou, Anna Amtmann, Michael R. Blatt

**Affiliations:** 1 Laboratory of Plant Physiology and Biophysics, Institute of Molecular, Cell and Systems Biology, Bower Building, University of Glasgow, Glasgow G12 8QQ, UK

**Keywords:** *Begonia*, gas exchange, photosynthesis, stomatal closure, WUE.

## Abstract

Stomata are microscopic pores formed by specialized cells in the leaf epidermis and permit gaseous exchange between the interior of the leaf and the atmosphere. Stomata in most plants are separated by at least one epidermal pavement cell and, individually, overlay a single substomatal cavity within the leaf. This spacing is thought to enhance stomatal function. Yet, there are several genera naturally exhibiting stomata in clusters and therefore deviating from the one-cell spacing rule with multiple stomata overlaying a single substomatal cavity. We made use of two *Begonia* species to investigate whether clustering of stomata alters guard cell dynamics and gas exchange under different light and dark treatments. *Begonia plebeja*, which forms stomatal clusters, exhibited enhanced kinetics of stomatal conductance and CO_2_ assimilation upon light stimuli that in turn were translated into greater water use efficiency. Our findings emphasize the importance of spacing in stomatal clusters for gaseous exchange and plant performance under environmentally limited conditions.

## Highlight

Non-contiguous clustering of small stomata in *Begonia* favours rapid stomatal closure by comparison with larger, solitary stomata, and enhances WUE under low light conditions.

## Introduction

Stomata are pores found in the epidermis of most aerial parts of plants and are formed between a specialized pair of cells, the guard cells. Stomata facilitate the uptake of CO_2_ at the expense of water vapour release via transpiration (Hetherington and Woodward, 2003). Hence, stomata control the trade-off between transpirational water loss and carbon gain, and therefore they play a crucial role in water use efficiency (WUE). Regulation of gas exchange is achieved by dynamically controlling the stomatal pore relative to environmental changes including light quality and intensity, ambient CO_2_ concentration, and humidity (Aphalo and Jarvis, 1991; Hetherington and Woodward, 2003; Shimazaki *et al.*, 2007). Stomatal movements are dependent on the transport and accumulation of osmotically active solutes (Blatt, 2000; Lawson and Blatt, 2014) as well as on the mechanistic properties of cells to allow lateral movements of guard cells (Franks and Farquhar, 2007).

Stomatal behaviour is also thought to be affected by the developmental pathway of stomatal lineage. The majority of plant species follow a ‘one-cell spacing rule’ during epidermal development that leads to the separation of stomata by at least one epidermal cell (Geisler *et al.*, 2000; Peterson *et al.*, 2010; Pillitteri and Dong, 2013). However, there are several genera that diverge from this rule, including members of the *Begonia* genus (Nebauer, 1967). Stomatal clustering in *Begonia* has been considered to be an adaptation for growth in ecological niches, imposing lower evaporative demand. For example, *Begonia heracleifolia* and *Begonia nelumbifolia* have been found to exhibit more stomatal clusters when growing on rocky surfaces near waterfalls rather than on well-watered soils (Hoover, 1986). In addition, Tang *et al.* (2002) reported that stomata in clusters share the same substomatal cavity and suggested a positive correlation between stomatal clustering and multiple layers of epidermis, which is regarded as a drought adaptation trait. No quantitative data are available confirming an advantage of species with stomatal clusters to grow in dry environments. To date, only studies with Arabidopsis transgenic lines have reported on the impact of stomatal clustering in leaf gas exchange and plant physiology (Dow *et al.*, 2014; Lehmann and Or, 2015; Papanatsiou *et al.*, 2016). Stomatal clustering in Arabidopsis mutants resulted in reduced stomatal conductance and assimilation of CO_2_ (Dow *et al.*, 2014), and in compromised movements of stomatal pores that were related to the altered ion transport in stomata found in clusters (Papanatsiou *et al.*, 2016).

We have revisited the physiological impact of stomatal clustering, by employing two species from the *Begonia* genus, *B. coccinea* and *B. plebeja*, the latter of which naturally forms stomatal clusters whereas the former does not (Burt-Utley, 1985). Stomatal clustering in *Begonia* differs from that of mutants of the model plant *Arabidopsis thaliana*. We find that stomatal clusters in *B. plebeja* are non-contiguous and therefore stomata are not in direct contact with each other despite occupying the same substomatal cavity. We also report on further morphological characteristics of stomata in *Begonia* species and their effects on stomatal behaviour. Our results emphasize the importance of spacing between stomata to allow plants to adjust gaseous exchange responses and enhance WUE in order to inhabit diverse niches.

## Materials and methods

### Plant material and growth conditions

*Begonia coccinea* and *Begonia plebeja* plants were obtained from Glasgow Botanic Gardens. Plants were grown under 70 μmol m^–2^ s^–1^ light in long-day conditions (16/8 h of light/dark), 22 °C/18 °C (light/dark) temperature, and 60%/70% (light/dark) relative humidity. Chemicals were reagent grade from Sigma-Aldrich.

### Gas exchange

Gas exchange was carried out using the LI-COR 6400 XT Infrared Gas Analyser (LICOR Biosciences) standard leaf chamber. Measurements were carried out at 22 °C, 60% relative humidity, and at 390 ppm CO_2_. Gas exchange responses were measured using an external light source (LI-COR 6400-18) and after leaves were adapted to the dark for 1.5 h. The spectral profile of the light source was adjusted to that of the growth rooms where plants were grown. At least three plants per species were measured on different days at the same point of the diurnal cycle. Data were normalized to a stomatal ratio of 0 since stomata in the *Begonia* species investigated are only found in the abaxial surface of the leaf.

### Stomatal assays

Stomatal patterns of *Begonia* plants were quantified by impressions taken from the abaxial area of mature young leaves. Xantopren VL Plus (silicon material, Heraeus, UK) and Activator (hardener, Heraeus, UK) were mixed in a ratio of 4:1, spread over the area of interest, and left to harden for at least 3 min before using a clear nail varnish to obtain the positive impression of the leaf epidermis.

Stomatal apertures were recorded from epidermal peels following pre-incubations in opening buffer (5 mM MES-NaOH pH 6.15, 60 mM KCl) for 2 h under 100 μmol m^–2^ s^–1^ light to open stomata fully. After imaging, epidermal peels were incubated for 5 min in depolarizing buffer supplemented with 20 μM fluorescein diacetate (FDA) and examined for fluorescence to confirm viability before the following further analysis as described before (Papanatsiou *et al.*, 2015).

Maximum stomatal opening, stomatal size, and density were estimated as described before (Papanatsiou *et al.*, 2016). Specifically, maximum stomatal aperture was estimated as

$$a_{\max}=\frac{\pi \times W_{a}\times L_{a}}{4}$$ (1)

where *W*_a_ is the aperture width and *L*_a_ is the aperture length.

Stomatal size was obtained from

$$Ss=\frac{\pi \times W_{s}\times L_{s}}{4}-a_{\max}$$ (2)

where *W*_s_ is the stoma width and *L*_s_ is the stoma length. The theoretical maximum stomatal conductance for water vapour (*G*_Wmax_) was calculated as described by Franks *et al.* (2009)

GWmaxd×SD×amaxv(lπ2amaxπ) (3)

where *d* is the diffusivity of water vapour in air (m^2^ s^–1^), *v* is the molar volume of air at 1 atm and 22 °C (m^3^ mol^–1^), SD is stomatal density (m^–2^), and *l* is the pore depth (m), estimated as the width of a fully inflated guard cell.

Stomatal closure was initiated after guard cells were fully open by superfusion with 10 mM MES-KCl, pH 6.1 supplemented with 6 mM CaCl_2_. Measurements were carried out on a cell-by-cell basis, and results are reported as means ±SE of *n* >80 stomata.

Both stomatal pattern and aperture were recorded by digital photomicrography using a Zeiss Axiovert200 microscope with Planapo ×20/0.80 objectives and an AxioCam HRc digital camera (Zeiss, Jena).

### Data and statistical analysis

Data analysis and curve fitting were carried out using SigmaPlot 12 (Systat Software Inc.). Statistical significance was determined using Student’s *t*-test at *P*<0.05.

## Results

**Fig. 1.**
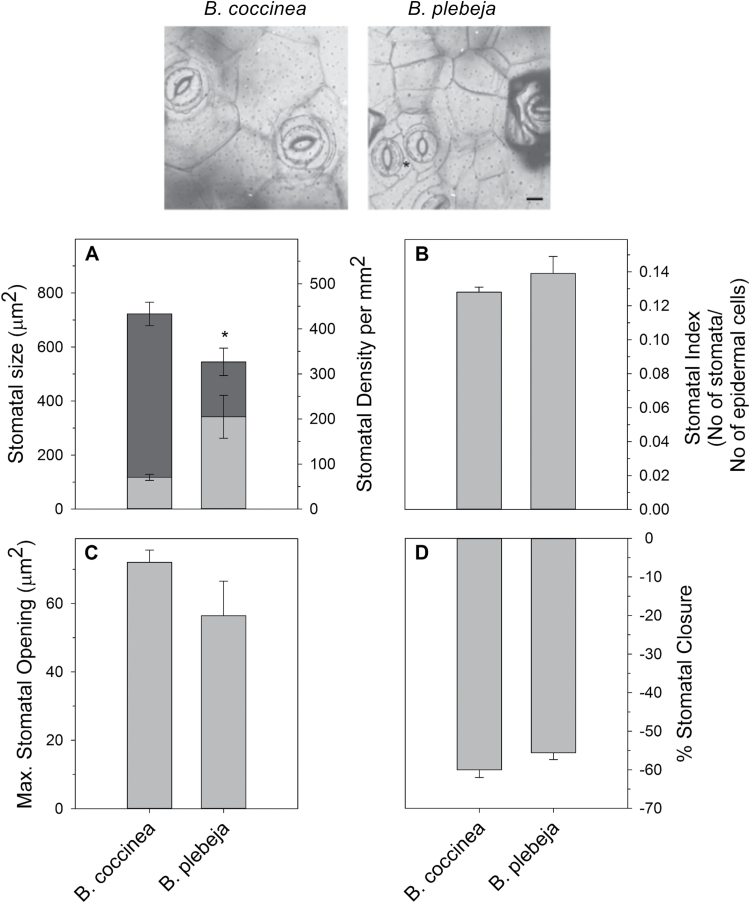
Stomatal characteristics of two *Begonia* species. The upper panel displays representative micrographs from the abaxial side of *B. coccinea* and *B. plebeja*. Scale bar=20 μm. Stomatal patterning was determined from epidermal peels of *B. coccinea* and *B. plebeja*. Graphs represent (A) stomatal density (light grey) and stomatal size (dark grey), (B) stomatal stomatal index, (C) maximum stomatal opening, and (D) percentage of stomatal closure relative to the maximum for that species. Data are means ±SE of *n* >60 stomata. The asterisk indicates a statistical difference (*P*<0.05), as determined by two-tailed *t*-test.

We employed two *Begonia* species with distinct stomatal patterning on *B. coccinea* and *B. plebeja* leaves (Fig. 1). Stomata in *B. plebeja* are found in clusters separated by a special type of non-stomatal cells (black asterisks; Fig. 1). In contrast, stomata in *B. coccinea* are solitary and surrounded by large epidermal cells. The mean stomatal density ranged from 70 to 200 stomata mm^–2^, with *B. plebeja* having significantly more stomata than *B. coccinea*. The stomatal size of *B. plebeja* was 24% smaller in comparison with *B. coccinea* (Fig. 1A). These data point to an inverse correlation between stomatal density and size that is in agreement with previous studies (Hunt and Gray, 2009; Doheny-Adams *et al.*, 2012; Papanatsiou *et al.*, 2015). However, when we calculated the stomatal index, which is the ratio of the number of stomata over the number of non-stomatal cells, no statistically significant differences between the two *Begonia* species were observed (Fig. 1B). The latter observation arises from the presence of the extra non-stomatal cells in stomatal clusters in *B. plebeja*.

**Fig. 2.**
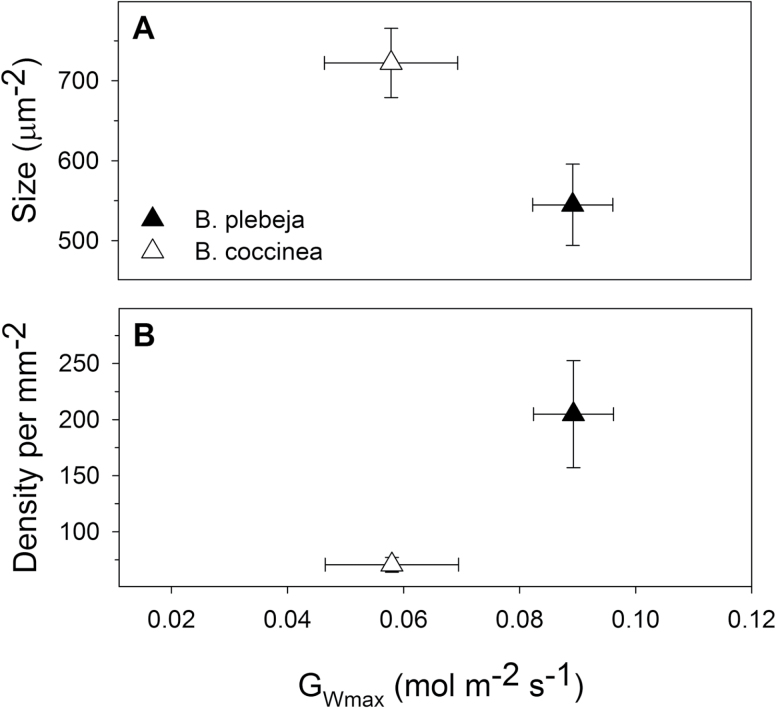
Relationship of maximum anatomical stomatal conductance to stomatal characteristics. Maximum anatomical stomatal conductance (*G*_Wmax_) was determined from stomatal geometry and maximum stomatal opening for *B. coccinea* (open triangles) and *B. plebeja* (filled triangles). Graphs represent the relationship of (A) stomatal size and (B) stomatal density to maximum anatomical stomatal conductance (*G*_Wmax_). Data are means ±SE of *n* >60 stomata.

Stomatal clustering has been shown to impact stomatal movements negatively in Arabidopsis (Papanatsiou *et al.*, 2016). We therefore examined stomatal opening in the two *Begonia* species by treating epidermal peels with opening buffer (60 mM KCl-MES, pH 6.1) under high light intensity for 2 h using standard protocols (Papanatsiou *et al.*, 2015). Maximum opening of stomatal pores was measured as described before (Doheny-Adams *et al.*, 2012; Papanatsiou *et al.*, 2016). Stomata of *B. plebeja* opened 22% less compared with *B. coccinea*, albeit that this difference was not statistically significant (Fig 1C). Similarly, no significant differences were observed when we subjected the epidermal peels from leaves of both *Begonia* species to closing buffer (10 mM KCl-MES+6 mM CaCl_2_, pH 6.1) and darkness for 90 min (Fig. 1D). Based on measurements of maximum stomatal opening and the geometry of stomata, we calculated the anatomical conductance to water vapour (*G*_Wmax_) of the two *Begonia* species according to Equation 1. *G*_Wmax_ describes the theoretical capacity of the leaf for gaseous exchange in relation to the total and maximum pore area (Franks and Beerling, 2009). The *G*_Wmax_ data agree with previous reports suggesting elevated *G*_Wmax_ in species having more stomata occupying the lead epidermis (Franks and Beerling, 2009). Indeed *B. plebeja* was estimated to have 34% greater *G*_Wmax_ when compared with *B. coccinea* plants (Fig. 2).

**Fig. 3.**
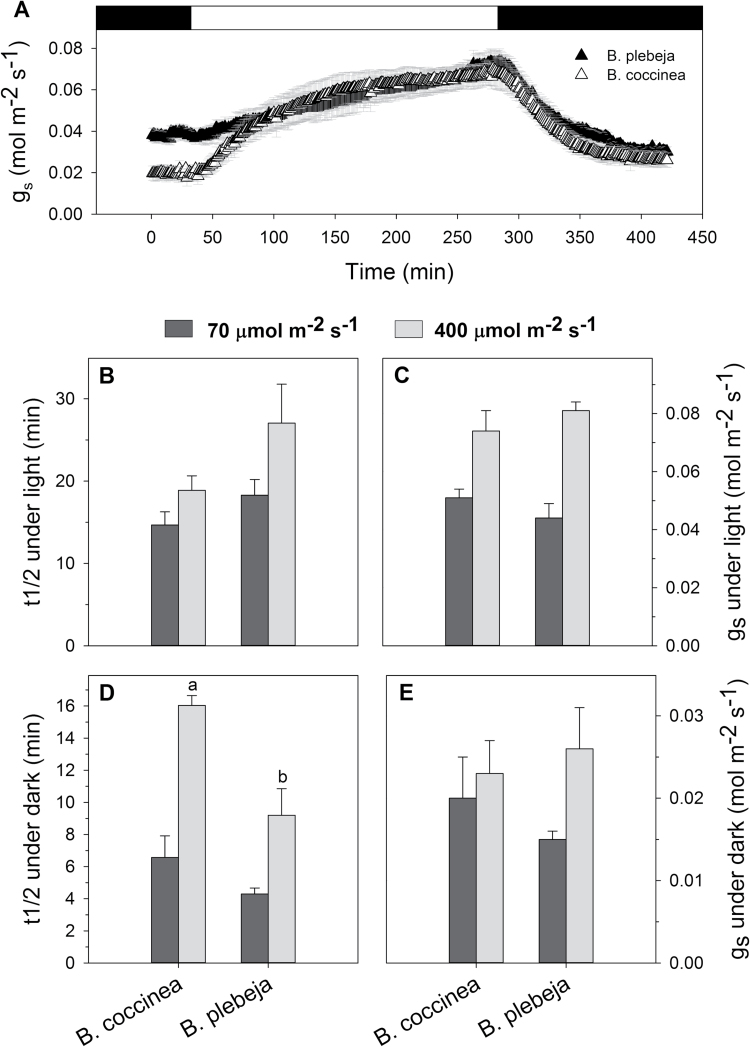
Stomatal patterning affects the gas exchange responses. (A) Representation of the experimental design measuring stomatal conductance (*g*_s_) response from dark-adapted leaves exposed to light and on subsequent transfer back to darkness. Graphs represent half-times (B and D) and steady-state rates (C and E) of *g*_s_ upon exposure to a light intensity of 70 μmol m^–2^ s^–1^ (dark grey) and 400 μmol m^–2^ s^–1^ (light grey) and on transfer back to darkness. The kinetics of gas exchange responses were extracted by separately fitting exponential function to the opening and closing response. Data are means ±SE of *n*=3 plants per species. Lower case letters indicate statistical differences (*P*<0.05), as determined by two-tailed *t*-test.

We also measured diffusive stomatal conductance (*g*_*s*_) from the *Begonia* species in response to light and dark treatments to investigate whether stomatal clustering of *B. plebeja* adversely influences gaseous exchange. *g*_*s*_ was measured from leaves of *B. coccinea* and *B. plebeja* plants that were dark adapted before exposure to a light intensity of either 70 μmol m^–2^ s^–1^ or 400 μmol m^–2^ s^–1^ and subsequently when transferred back to darkness (Fig. 3A). We determined the kinetics of *g*_*s*_ responses in light and dark treatments via non-linear curve fitting to either the opening or closing response (Fig. 3). The steady-state *g*_*s*_ values in the dark and each of the light regimes were statistically indistinguishable between *B. coccinea* and *B. plebeja* (Fig. 3C, E). Stomata of *B. coccinea* and *B. plebeja* responded at a similar speed upon exposure to a light intensity of 70 μmol m^–2^ s^–1^ and on the subsequent transfer to darkness. Similarly, exposure of leaves to high light intensity did not result in any significant difference in the opening half-times of *g*_*s*_ of *B. coccinea* and *B. plebeja*, with those being 19 ± 2 min and 27 ± 4 min, respectively. Interestingly, we noted that *B. plebeja* responded 42% faster in closure response when the leaves were transferred from high light back to darkness.

**Fig. 4.**
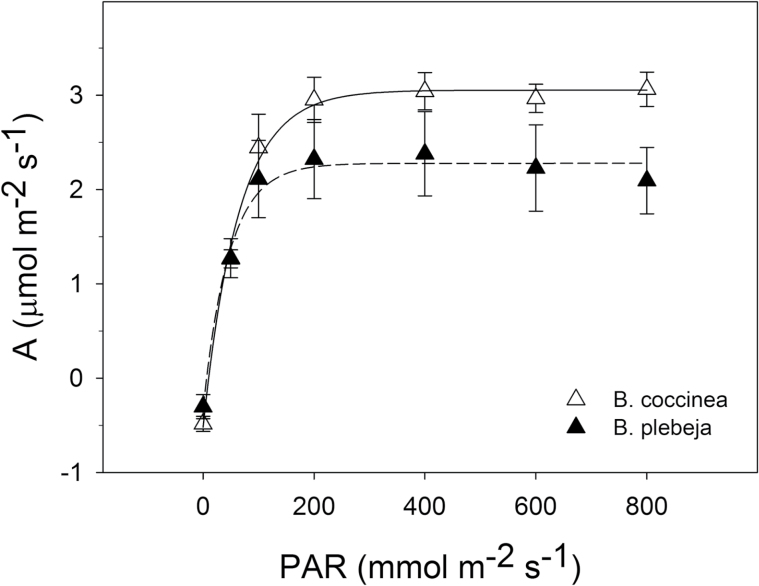
Effect of photosynthetic active radiation on CO_2_ assimilation. Light curves from *B. coccinea* (open triangles) and *B. plebeja* (filled triangles) display the assimilation of CO_2_ over a series of quantum flux densities ranging from 0 to 800 μmol m^–2^ s^–1^. Data were jointly fitted to an exponential rise curve, and fits are shown for *B. coccinea* (solid line) and *B. plebeja* (dashed line). Data are means ±SE of *n*=3 plants per species.

We carried out analysis of light responses to determine the steady-state assimilation rate of CO_2_ (*A*) over a range of distinct quantum flux densities (Fig. 4). Both *B. coccinea* and *B. plebeja* responded similarly to increasing photosynthetic active radiation. The CO_2_ assimilation response followed an exponential rise and reached a maximum rate after leaves were exposed to 200 μmol m^–2^ s^–1^ of light. The CO_2_ assimilation rates were undifferentiated between the two *Begonia* species at light intensities <400 μmol m^–2^ s^–1^. In contrast, *B. plebeja* showed smaller CO_2_ assimilation rates in comparison with those of *B. coccinea* at saturating light intensities.

We also performed *A*/*C*_i_ curves where *A* was estimated over a range of CO_2_ concentrations (see Supplementary Fig. S1 at *JXB* online). Fitting a non-linear regression model to the *A*/*C*_i_ curves allows us to distinguish among three distinct parameters contributing to photosynthetic machinery: the carboxylation rate of Rubisco (*V*_cmax_); the electron transport (*J*); and the triose phosphate use (TPU) reactions (Sharkey *et al.*, 2007). All three photosynthesis-related biochemical reactions were statistically indistinguishable between the two *Begonia* species at the lower light intensities.

**Fig. 5.**
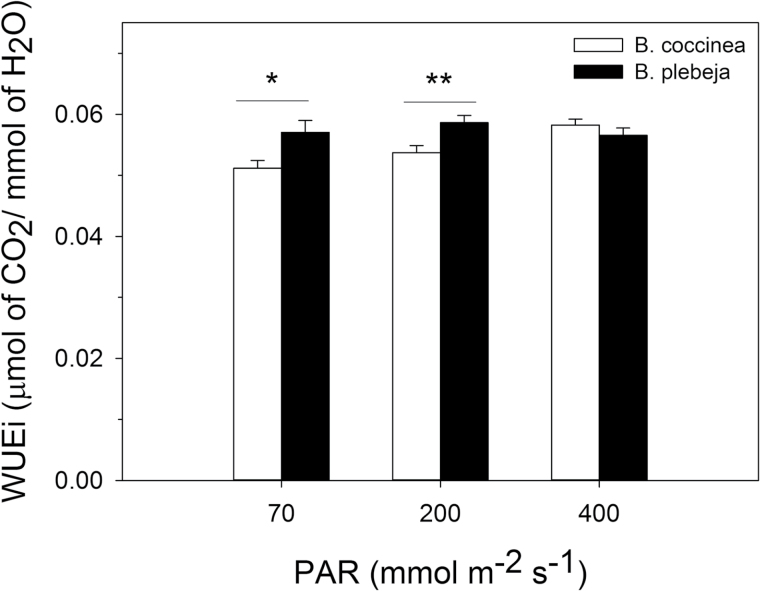
Intrinsic water use efficiency (WUE_i_) of *Begonia* plants under three light regimes. The WUE_i_ of *B. coccinea* (white bars) and *B. plebeja* (black bars) was estimated as the ratio of the maximum CO_2_ assimilation rate over stomatal conductance at a light intensity of 70, 200, and 400 μmol m^–2^ s^–1^. Data are means ±SE of *n*=3 plant per species. Asterisks indicate statistically significant differences (*P*<0.05) between the two *Begonia* species at each light intensity, as determined by two-tailed *t*-test.

The above-mentioned differences in CO_2_ assimilation prompted us to investigate whether the distinct stomatal patterning and behaviour of the two *Begonia* species would influence WUE depending on the light regime. We therefore measured the intrinsic WUE (WUE_i_), calculated here as the ratio of the CO_2_ assimilation rate and stomatal conductance at 70, 200, and 400 μmol m^–2^ s^–1^ of light (Fig. 5). To our surprise, *B. plebeja* plants appear to show an increase in the WUE_i_ of ~30% compared with *B. coccinea* plants at light intensities of 70 μmol m^–2^ s^–1^ and 200 μmol m^–2^ s^–1^. Yet, at the saturating light intensity, WUE_i_ was statistically undifferentiated between the two species.

## Discussion

The reduction in diffusive capacity of stomata found in clusters has recently been uncovered through studies employing mutants of the model organism Arabidopsis that show contiguous stomatal clusters [i.e. stomata are in direct contact (Dow *et al.*, 2014; Lehmann and Or, 2015; Papanatsiou *et al.*, 2016)]. Yet, species naturally having altered stomatal patterning, such as *B. plebeja*, display non-contiguous stomatal clusters (see Fig. 1). This unique morphological property of *B. plebeja* makes it a great tool to enrich further the selection of plants with different stomatal spatial arrangements, and therefore characterize how the mechanistic properties of stomata influence leaf gaseous exchange. We note, however, that *B. coccinea* and *B. plebeja* differ in many genetic and other morphological aspects and therefore we do not conclude that their difference in stomatal morphology is the sole driving force for their different adaptive strategies.

To the extent that *B. plebeja* shows non-contiguous stomatal clusters, it resembles Arabidopsis mutants with high numbers of small satellite and solitary stomata (Doheny-Adams *et al.*, 2012; Dow *et al.*, 2014; Hepworth *et al.*, 2015). Reducing the size of the guard cells surrounding the stomatal pore has the effect of increasing the ratio of membrane surface area to guard cell volume. Provided the density of membrane transporters per unit surface area is nearly constant, a decrease in guard cell size can be expected to accelerate the solute flux per unit volume proportionally, thereby allowing for faster responses to environmental transients. This strategy is well documented in studies of Arabidopsis (Schlüter *et al.*, 2003; Tanaka *et al.*, 2013) and other plant species (Franks and Farquhar, 2007; Drake *et al.*, 2013; Lawson and Blatt, 2014), and it is consistent with mathematical models that take into account the geometry of guard cells and the guard cell complex (Doheny-Adams *et al.*, 2012; Dow *et al.*, 2014, Lehman and Or, 2015) as well as membrane transport (Chen *et al.*, 2012; Wang *et al.*, 2012; Lawson and Blatt, 2014). Indeed, we observed a faster closing response of *B. plebeja* leaves, especially when this was commenced from a low light intensity (see Fig. 3).

Under lower light, gaseous exchange and therefore WUE is dependent on the light-limited photosynthetic reactions, whereas at high light intensities the WUE is mainly controlled by stomatal conductance (Lawson, 2009; Hummel *et al.*, 2010; Eisenach *et al.*, 2012; Lawson and Blatt, 2014). Thus, one can speculate that the enhanced dynamic movements of stomata together with the elevated WUE at limited photosynthetic conditions could be advantageous to the performance of the species. Reports on the habitat of *B. plebeja* suggest that this species is often found in rocks close to waterfalls, which limit water supply due to low water retention, and at the lower levels of forest canopy (Tebitt, 2005). In addition, Hoover (1986) suggested that stomatal clustering in *Begonia* species is an adaptive strategy to water-restricted environments. The altered stomatal patterning has previously been shown to affect gas exchange responses in Arabidopsis (Schlüter *et al.*, 2003; Franks *et al.*, 2009; Dow *et al.*, 2014); high stomatal density resulted in an increase in CO_2_ assimilation (Tanaka *et al.*, 2013), while the decrease in stomatal number resulted in the reduction of transpirational water loss (Yoo *et al.*, 2010; Doheny-Adams *et al.*, 2012). It is therefore suggested that stomatal patterning might provide a tool for fine-tuning the trade-off between these two processes, thus improving WUE (Drake *et al.*, 2013; Lawson and Blatt, 2014).

Collectively, the data describe the difference in the spatial arrangement of stomata in the two *Begonia* species, and point to an unaltered dynamic range of movement of stomata found in clusters compared with those appearing solitary. Most importantly, we argue that the non-contiguous clustering of small stomata is a favourable trait over solitary large stomata by virtue of the faster stomatal closing response and the enhanced WUE_i_, especially under low light conditions. We speculate that high numbers of small stomata residing over the same substomatal cavity can confer an advantage to plants subjected to low water regimes. Undoubtedly, future comparative studies employing natural and experimental plant populations with distinct stomatal patterning should elucidate the above speculation and assess the impact of stomatal clustering in plant adaptation and performance.

## Supplementary data

Supplementary data are available at *JXB* online.

Fig. S1. *A*/*C*_i_ curves of *B. coccinea* and *B. plebeja* plants under two light regimes.

## Authors contribution

MP carried out the gas exchange measurements and stomatal assays; MP and MRB analysed the data; MP, AA, and MRB wrote the article.

## Supplementary Material

supplementary_figure_S1
