# Supplementary material for: Stomatal clustering in Begonia associates with the kinetics of leaf gaseous exchange and influences water use efficiency
Source: J Exp Bot. 2017 Mar 28;68(9):2309–15. doi: 10.1093/jxb/erx072 (PMC5447881; doi:10.1093/jxb/erx072)
Supplement: supplementary_figure_S1 [file erx072_suppl_supplementary_figure_S1.pdf]

Title: Stomatal clustering in *Begonia* associates with the kinetics of leaf gaseous exchange and influences water use efficiency

Maria Papanatsiou, Anna Amtmann and Michael R. Blatt

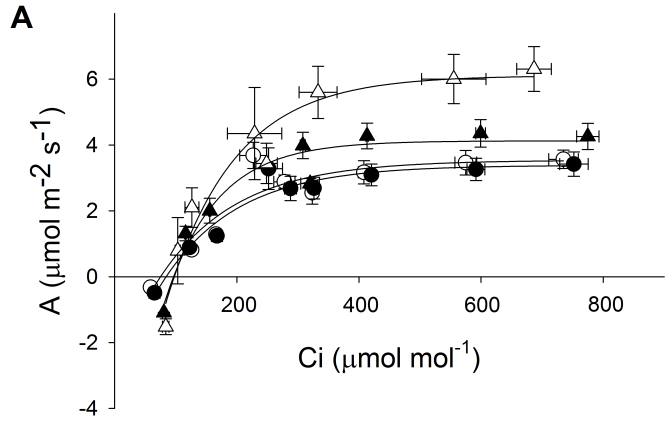

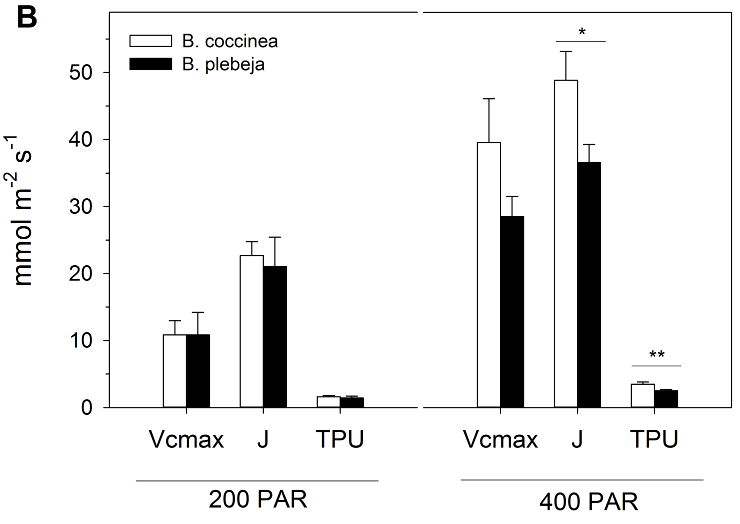

Suppl. Fig1. A/Ci curves of *B. coccinea* and *B. plebeja* plants under two light regimes.

(A) A/Ci curves of B. coccinea (white symbols) and B. plebeja (black symbols) were estimated at 200 (circles) or 400 (triangles) μmol m^{-2} s^{-1}. Data are means ±SE of n=3 plant per species.

(B) Three components of photosynthetic machinery contributing to CO2 assimilation were estimated by fitting a non-linear model as (SHARKEY et al., 2007). Vcmax corresponds to the carboxylation rate of Rubisco, J to the electron transport and TPU to the triose phosphate use. Data are means ±SE of n=3 plant per species. Asterisks indicate statistically significant differences ($P<0.05$) between the two Begonia species at each light intensity, as determined by t-test.
